# Caregiver Perspectives on Patient Participation in Biological Pediatric Cancer Research

**DOI:** 10.3390/children9060901

**Published:** 2022-06-16

**Authors:** Nicole E. Kendel, Jennifer A. Belsky, Joseph R. Stanek, Keri A. Streby, Nilay Shah

**Affiliations:** 1 Division of Pediatric Hematology/Oncology, Nationwide Children’s Hospital, The Ohio State University, Columbus, OH 43205, USA; nicole.kendel@nationwidechildrens.org (N.E.K.); jbelsky@iu.edu (J.A.B.); joseph.stanek@nationwidechildrens.org (J.R.S.); keri.streby@nationwidechildrens.org (K.A.S.); 2 Riley Hospital for Children, Indiana University, Indianapolis, IN 46202, USA; 3 Biostatistics Resource at Nationwide Children’s Hospital, Columbus, OH 43205, USA

**Keywords:** biological sampling, pediatric, oncology, cancer, research

## Abstract

Adolescent cancer patients and their caregivers have demonstrated willingness to participate in invasive biological sampling, either for their own potential benefit or for research purposes. However, many malignancies occur primarily in prepubescent patients and there are no similar studies in this population. Our study objective was to assess the willingness of caregivers to consent to research studies involving invasive biological sampling in children ≤ 13 years of age. Participants completed a survey assessing their willingness to allow various procedures both with and without clinical benefit to their children. Most respondents were willing to allow additional blood draws regardless of potential benefit to their children (95.6% were willing when there would be benefits and 95.6% were willing when there would not). Although the overall willingness was lower with other hypothetical procedures, the majority of respondents were still willing to allow additional biopsies for research purposes. Caregivers of young children with cancer will allow their children to undergo additional invasive procedures for research purposes. This willingness decreased with more invasive procedures without potential direct benefit, but interest remained in more than half of participants. Caregivers for young patients with cancer should be approached for participation in future biological/correlative studies.

## 1. Introduction

Advances in the treatment of pediatric cancers have improved the five-year overall survival rates from just over 60% in 1975 to approximately 80% in 2002 [1]. This progress has been considerably bolstered through the study of patients’ blood, bone marrow, and tumor tissue, obtained at initial diagnosis. These biological samples have enhanced our understanding of pediatric cancer biology and subsequently informed improvements in first-line treatment. Unfortunately, treatment of recurrent malignancies remains a major clinical and scientific challenge, partly due to a relative paucity of samples being obtained at relapse, limiting the study of the pathophysiology of recurrent disease.

Historically, refractory or recurrent primary tumors and metastases were not routinely biopsied because (1) additional biopsies were not thought to be clinically beneficial, and (2) concerns about patients’ and caregivers’ possible reluctance to allow invasive procedures for research purposes discouraged tissue acquisition. However, studies have shown that refractory and recurrent tumors are often genetically distinct from the primary tumors, involving activation of different oncogenic processes compared to those driving initial tumorigenesis [2,3]. Current genomic methods now allow for deeper tumor assessment, increasing the potential for new clinically relevant findings and research directions [4,5]. The characterization of refractory or relapsed disease may allow practitioners to adapt treatment approaches and improve disease-free survival rates while also advancing research into new directions. Advances in minimally invasive biopsy techniques have significantly reduced the risks associated with surgical procedures, potentially making tissue sampling more palatable to families [6,7]. However, patient and caregiver viewpoints on such procedures remain widely unknown.

Studies have shown that adolescent cancer patients and their caregivers are willing to participate in invasive biological sampling, either for their own potential benefit or for research purposes [8,9]. However, many malignancies occur primarily in prepubescent patients, with few studies specifically investigating the willingness of this unique patient population. As such, we surveyed caregivers of prepubescent pediatric oncology patients to assess their willingness to consent to research studies involving invasive biological sampling. We specifically evaluated whether their viewpoints were affected by the degree of invasiveness and the potential of clinical benefit for their children. We hypothesized that caregivers would be willing to allow their children to provide both blood and tissue samples for oncology research, regardless of benefit to their children.

## 2. Materials and Methods

This single-institution study evaluated caregivers of patients with oncologic diagnoses receiving treatment or follow-up care at Nationwide Children’s Hospital in Columbus, Ohio, from July 2019 to December 2020. Caregivers were eligible if they (1) spoke English as their primary language and (2) were the primary caregivers of patients with current or historical cancers that were diagnosed at ≤13 years of age. Subjects were identified by a clinician team member via electronic medical records and at team meetings. There was no compensation provided for participation.

The caregiver survey tool was developed by pediatric oncologists, psychologists, education specialists, and statisticians (Table S1). The survey was approved for its ease of understanding and content at a third-grade reading level. Several concepts and questions were adapted from a previously published study [10]. Participants were asked about their willingness to allow various procedures when there was a possibility of clinical benefit to their child and when there was no chance of benefit. Survey responses were measured on a 5-point Likert scale, ranging from “extremely unwilling” to “extremely willing”. The survey was administered by a trained study team member and responses were stored securely in a REDCap database.

All responses were de-identified, compiled, and analyzed. Demographic information was summarized using descriptive statistics, and survey responses were presented as frequencies and percentages or median and range or interquartile range (IQR). Unpaired Wilcoxon rank sum and paired signed rank tests were used to compare the difference in caregiver willingness to agree with additional procedures for research purposes based on whether there was potential benefit to their child or not. The *p*-values were two-sided and those less than 0.05 were considered statistically significant. Statistical analyses were completed using the base R statistical package. The research protocol was reviewed and approved by the Institutional Review Board at Nationwide Children’s Hospital (FWA00002860).

## 3. Results

A total of 48 caregivers were approached and 46 caregivers completed the survey for 46 unique patients (Table 1). If multiple caregivers were present, only one caregiver completed the survey. All respondents reported that their children were diagnosed at 13 years of age or younger, with a median diagnosis age of 3.5 years (range 0–13 years). The mean length of time from diagnosis to survey completion was 1.2 years (range 0–9). Most respondents identified as a parent, while one person identified as a grandparent. Levels of education varied, with 32.6% reporting a bachelor’s degree, 23.9% reporting a high school degree or general education development equivalent, and 17.4% reporting some college education. Over half of respondents reported some type of employment (56.6%), while 8.7% reported that they were out of work and an additional 13.0% reported that they were unable to work.

In our assessment of willingness to consent to biological sampling, most respondents were willing to allow additional blood draws, regardless of whether those blood draws would benefit their child (95.6% were willing when there would be benefits and 95.6% were willing when there would not) (Table 2). Overall willingness was lower with other hypothetical procedures. Two-thirds of caregivers were willing to allow an extra bone marrow biopsy and aspirate if it provided benefit to their child, compared to 58.3% when it did not provide benefit. Respondents were willing to allow an additional image-guided biopsy of the primary tumor during treatment, with 81.5% willing when it provided benefit compared to 60.7% of responses when it did not provide benefit. Comparatively, caregiver’s willingness to allow an image-guided biopsy in the presence of metastatic disease was reported in 79.3% of respondents when it provided benefit compared to 56.7% of respondents when it did not provide benefit.

Initial unpaired comparisons did not reveal any statistically significant differences in willingness scores based on benefit to the child (Table 3). In contrast, a paired analysis directly comparing each individual caregiver’s willingness for procedures revealed significantly lower willingness scores for more invasive procedures (bone marrow biopsy/aspirate, image-guided biopsy, and image-guided biopsy in the presence of metastatic disease) that did not provide benefit to their child. There was no significant difference in willingness scores for additional blood draws. Despite these differences, most respondents were still willing to allow additional bone marrow and tumor biopsies for research purposes, regardless of benefit to their child.

## 4. Discussion

This study is the first to examine the willingness of caregivers for prepubescent oncology patients to undergo additional invasive procedures for research purposes. In this single-center cohort, most caregivers were receptive to additional invasive biological procedures on their children. Although willingness to participate was lower for more invasive procedures without expected benefit, compared to those with potential benefit, more than half of the respondents were still willing to participate.

The majority of the current literature regarding willingness to consent for invasive research procedures in pediatric patients focuses on the adolescent population. These prior studies revealed an overall willingness to undergo additional invasive sampling, regardless of clinical benefit to the participant [8,9]. Caregivers expressed some discomfort with agreeing to additional procedures, but adolescents were comparatively more willing to participate in invasive research [9,11]. Adolescents who had been diagnosed more than 6 months before the time of survey were more willing to undergo invasive procedures for research purposes [8]. There are multiple speculations regarding this finding, including a comparatively increased focus on treatment for those with new diagnoses and the potential for increased trust and appreciation for research in those who are already undergoing treatment. Because the mean length of time from diagnosis was 1.2 years in our study, caregiver responses may have been influenced by these same theories. The only published study evaluating caregiver perceptions about prepubescent patients found that the willingness to participate in directly nonbeneficial research studies depended on the potential risks of such studies, with reduced participation as the perceived risk increased [12]. This study excluded patients younger than 7 years of age, and it did not ask about specific procedures such as those examined in our study.

Studies examining pediatric oncology patients’ and caregivers’ viewpoints on research participation have been more limited in number and did not evaluate perspectives on invasive procedures. One single-center study revealed that most patients found it difficult to understand the terms used in the assent process and half of patients did not realize that their treatments were considered research [13]. Most patients wanted to participate in research to help other children, but also thought that the treatments they were receiving were the best options for their diseases, rather than seeing them as part of the studies [13]. Several end-of-life studies in adolescent oncology patients have also shown that parents and children alike choose to participate in research studies due to a desire to help others and prevent the experience of similar suffering [14,15]. In fact, when surveying caregivers of patients who had died as a result of their oncologic processes, none of the caregivers rescinded their consent to participate in research studies [15].

Our results are limited by the use of a voluntary survey at a single institution, leading to a small sample size and the potential selection bias of participants that may not be representative of the general population of pediatric oncology patients. Patients were also selected at variable and unidentified treatment timepoints, which may have influenced caregiver responses. We chose not to evaluate the viewpoints of the prepubescent patients because less than a quarter of the patients enrolled in our study were considered eligible for assent. Performing a similar study with a larger sample size across multiple institutions facilitates the evaluation of these patients’ perspectives as well as caregivers’ understanding of the procedures and any additional factors that may affect their choices to participate.

Our results demonstrate that caregivers of young children with cancer will allow their children to undergo additional invasive procedures for research purposes. This willingness decreases with procedures that do not provide potential direct benefit, but the interest remained in more than half of the participants. These viewpoints are particularly important given the growing need for patient-derived blood and tissue specimens to further advance current knowledge in pediatric cancer treatments. These viewpoints should be considered when designing future biological/correlative studies. Caregivers for young patients with cancer should be approached for participation in these necessary clinical trials, with best efforts to clarify the purposes, risks, and benefits of all procedures and treatment.

## Figures and Tables

**Table 1:** Demographic information for caregivers completing the survey tool.

Characteristic	N (%)
Total respondents	46
Median age, years (range)	34.5 (21–71)
**Relation to Child**	
Parent	45 (97.8)
Grandparent	1 (2.2)
**Gender**	
Male	10 (21.7)
Female	32 (69.6)
Unknown	4 (8.7)
**Race ^a^**	
White	43 (93.5)
Black	4 (8.7)
Latino/Hispanic	1 (2.2)
Asian/Pacific Islander	1 (2.2)
**Marital Status**	
Single, never married	10 (21.7)
Married or domestic partnership	34 (73.9)
Divorced	2 (4.3)
**Highest Education**	
Some high school	2 (4.3)
High school/GED	11 (23.9)
Some college	8 (17.4)
Trade/vocational training	3 (6.5)
Associate degree	4 (8.7)
Bachelor’s degree	15 (32.6)
Master’s degree	1 (2.2)
Professional degree	2 (4.3)
**Employment Status**	
Employed for wages	21 (45.7)
Self-employed	5 (10.9)
Out of work	4 (8.7)
Homemaker	9 (19.6)
Retired	1 (2.2)
Unable to work	6 (13.0)
Median age of child at diagnosis, years (range)	3.5 (0–13)
Median age of child at survey, years (range)	5.0 (0–13)
Mean length of time between diagnosis and survey completion, years (range)	1.2 (0–9)

^a^ Three caregivers selected both White and Black as their ethnicity.

**Table 2:** Summary of caregiver survey responses regarding additional procedures that either provide or do not provide benefit to their child.

Question	Number of Responses, N	Willing ^a^, N (%)	Unwilling ^b^, N (%)	Neutral, N (%)
As a caregiver, how willing are you to allow:				
Additional blood draws that may help your child’s health	46	44 (95.6)	2 (4.3)	0 (0)
Additional blood draws that would help other children	46	44 (95.6)	2 (4.3)	0 (0)
Extra bone marrow biopsy and aspirate to find cancer cells or improve your child’s treatment	39 ^c^	26 (66.7)	4 (10.2)	9 (23.1)
Extra bone marrow biopsy and aspirate to help other children with cancer	36 ^c^	21 (58.3)	6 (16.7)	9 (25.0)
Image-guided biopsy of primary tumor during treatment that may test how well treatment is working or otherwise improve your child’s health	27 ^d^	22 (81.5)	2 (7.4)	3 (11.1)
Image-guided biopsy of primary tumor during treatment to help other children with cancer	28 ^d^	17 (60.7)	6 (21.4)	5 (17.9)
If metastatic disease, image-guided biopsy that may test how well treatment is working or otherwise improve your child’s health	29 ^d^	23 (79.3)	3 (10.3)	3 (10.3)
If metastatic disease, image-guided biopsy to help other children with cancer	30 ^d^	17 (56.7)	7 (23.3)	6 (20)

^a^ Willingness defined by “willing” or “extremely willing” responses to the survey question. ^b^ Unwillingness defined by “unwilling” or “extremely unwilling” responses to the survey question. ^c^ Respondents were asked not to respond if their child had not previously undergone a bone marrow aspirate and/or biopsy, leading to a smaller number of responses. ^d^ Respondents were asked not to respond to this survey question if their child either had leukemia or had a total resection of their tumor at diagnosis, leading to a smaller number of responses.

**Table 3:** Comparisons of survey responses based on benefit to their child.

Procedure	With Benefit	Without Benefit
**Additional Blood Draws**		
N	46	46
Median (interquartile range) ^a^	5 (4–5)	5 (4–5)
Mean (standard deviation) ^a^	4.61 (0.80)	4.41 (0.88)
p (unpaired) ^b^	0.10	
p (paired) (N = 46) ^c^	0.14	
**Extra Bone Marrow Biopsy**		
N	39	36
Median (interquartile range) ^a^	4 (3–5)	4 (3–4)
Mean (standard deviation) ^a^	3.85 (1.04)	3.56 (1.03)
p (unpaired) ^b^	0.21	
p (paired) (N = 36) ^c^	0.0346	
**Image-Guided Biopsy**		
N	27	28
Median (interquartile range) ^a^	4 (4–5)	4 (3–4)
Mean (standard deviation) ^a^	4.00 (0.96)	3.54 (1.11)
p (unpaired) ^b^	0.09	
p (paired) (N = 26) ^c^	0.0010	
**Image-Guided Biopsy If Metastatic Disease**		
N	29	30
Median (interquartile range) ^a^	4 (4–4.5)	4 (2.8–4)
Mean (standard deviation) ^a^	3.86 (1.06)	3.40 (1.13)
p (unpaired) ^b^	0.08	
p (paired) (N = 29) ^c^	0.0027	

^a^ Scale: 1 = extremely unwilling, 2 = unwilling, 3 = neutral, 4 = willing, 5 = extremely willing. ^b^ Unpaired *p*-values result from Wilcoxon rank sum tests and compare “with benefit” and “without benefit” responses as independent groups, allowing for the inclusion of all data. ^c^ Paired *p*-values result from signed rank tests and compare “with benefit” and “without benefit” responses for each individual caregiver, requiring caregivers to respond to both questions, leading to smaller sample sizes.

## Data Availability

The data presented in this study are available on request from the corresponding author.

## Supplementary Materials

The following supporting information can be downloaded at: https://www.mdpi.com/article/10.3390/children9060901/s1, Table S1: Caregiver perspectives survey tool.
