# Supplementary material for: Caregiver Perspectives on Patient Participation in Biological Pediatric Cancer Research
Source: Children (Basel). 2022 Jun 16;9(6):901. doi: 10.3390/children9060901 (PMC9221797; doi:10.3390/children9060901)
Supplement: Supplementary file 1 [file children-09-00901-s001.zip › children-1744609-supplementary.pdf]

Table S1. Caregiver perspectives survey tool.

<table>
<tr><th>Question</th><th>Answer Choices</th></tr>
<tr><td>1. How willing would you be to allow additional blood[a] draws during treatment for research that may potentially help your child’s health, including looking for any remaining cancer cells?</td><td rowspan="8">A. Extremely Unwilling
B. Unwilling
C. Neutral
D. Willing
E. Extremely Willing
F. Not applicable</td></tr>
<tr><td>2. How willing would you be to allow an extra bone marrow biopsy and aspiration[b] for research, with the possibility to find cancer cells or improve your child’s treatment?</td></tr>
<tr><td>3. How willing would you be to allow image-guided biopsies[c] to be obtained for research at different times during treatment, before your child’s tumor was fully removed, to potentially test how well the treatments were working or otherwise improve their treatment?</td></tr>
<tr><td>4. If your child’s tumor had spread to other parts of his or her body (metastasis), how willing would you be to allow image-guided biopsies[c] of one or more metastases as well as the original tumor to be obtained for research at different times during treatment, to potentially test how well the treatments were working or to otherwise improve their health?</td></tr>
<tr><td>5. How willing would you be to allow additional blood draws[a] during treatment for research that would help the treatment of children diagnosed with cancer in the future?</td></tr>
<tr><td>6. How willing would you be to allow an extra bone marrow biopsy and aspiration[b] for research, to help the treatment of children diagnosed with cancer in the future?</td></tr>
<tr><td>7. How willing would you be to allow image-guided biopsies[c] to be obtained for research at different times during treatment, before your child’s tumor was fully removed, to potentially help the treatment of children diagnosed with cancer in the future?</td></tr>
<tr><td>8. If your child’s tumor had spread to other parts of his or her body (metastasis), how willing would you be able to allow image-guided biopsies[c] of one or more metastases as well as the original tumor to be obtained for research at different times during the treatment, to potentially help the treatment of children diagnosed with cancer in the future?</td></tr>
<tr><td colspan="2">[a] Additional blood draws were described as “1-2 teaspoons of blood. The amount of blood taken would not harm your child.”
[b] Bone marrow biopsy and aspiration were described with the following statement: “This procedure would be done under sedation (your child would be asleep under anesthesia) as an outpatient procedure. This is a standard procedure performed regularly on patients. While there would be some potential risk (bleeding or infection) these would be unlikely. Your child may feel some pain for a day that can usually be controlled with Tylenol. Your child would be expected to return to normal activity within a day.”
[c] Image-guided biopsies were described with the following statement: “This type of procedure would be done under sedation (your child would be asleep under anesthesia). A CT scan or ultrasound would be used by a radiologist to find the tumor; the radiologist would then use a small needle, the size of a thin straw, to go through the skin and take samples from the tumor. This would be an outpatient procedure. This is a standard procedure performed regularly on patients. While there would be some potential risk (bleeding or infection) these would be unlikely. Your child may feel some pain for a day that can usually be controlled with tylenol. Your child would be expected to return to normal activity within a day.”</td></tr>
</table>
